# Machine learning-based predictive modelling for the enhancement of wine quality

**DOI:** 10.1038/s41598-023-44111-9

**Published:** 2023-10-09

**Authors:** Khushboo Jain, Keshav Kaushik, Sachin Kumar Gupta, Shubham Mahajan, Seifedine Kadry

**Affiliations:** 1 School of Computer Science, University of Petroleum and Energy Studies, Dehradun, India; 2 Department of Electronics and Communication Engineering, Central University of Jammu, Samba, Jammu, Jammu and Kashmir 181143 India; 3 Hourani Center for Applied Scientific Research, Al-Ahliyya Amman University, Amman, Jordan; 4 University Center for Research & Development (UCRD), Chandigarh University, Mohali, India; 5 Department of Applied Data Science, Noroff University College, Kristiansand, Norway; 6 Department of Electrical and Computer Engineering, Lebanese American University, Byblos, Lebanon

**Keywords:** Electrical and electronic engineering, Engineering

## Abstract

The certification of wine quality is essential to the wine industry. The main goal of this work is to develop a machine learning model to forecast wine quality using the dataset. We utilised samples from the red wine dataset (RWD) with eleven distinct physiochemical properties. With the initial RWD, five machine learning (ML) models were trained and put to the test. The most accurate algorithms are Random Forest (RF) and Extreme Gradient Boosting (XGBoost). Using these two ML approaches, the top three features from a total of eleven features are chosen, and ML analysis is performed on the remaining features. Several graphs are employed to demonstrate the feature importance based on the XGBoost model and RF. Wine quality was predicted using relevant characteristics, often referred to as fundamental elements, that were shown to be essential during the feature selection procedure. When trained and tested without feature selection, with feature selection (RF), and with key attributes, the XGBoost classifier displayed 100% accuracy. In the presence of essential variables, the RF classifier performed better. Finally, to assess the precision of their predictions, the authors trained an RF classifier, validated it, and changed its hyperparameters. To address collinearity and decrease the quantity of predictors without sacrificing model accuracy, we have also used cluster analysis.

## Introduction

**Figure 1:**
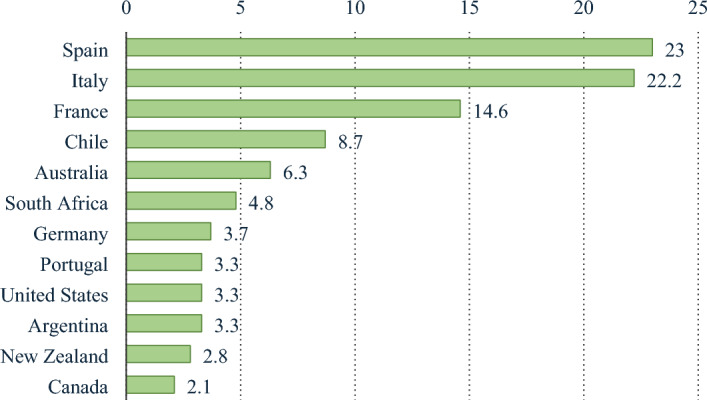
Wine export volume in a million hectolitres in 2021.

Today, a wider group of customers is enjoying wine more and more. In 2021, wine exports from all nations reached a global total of $40.7 billion. Since 2017, when wine shipments were valued at $35.4 billion, that amount in dollars represents an average 15% growth for all exporting countries^1^. Export sales of wines increased by 19.8% from $34.3 billion in 2020 annually. France, Italy, Spain, Chile, and Australia are the top five countries for wine exports (Fig. 1). Regarding dollar sales, that potent group of suppliers was responsible for 70.4% of the wine exported worldwide. With shipments totalling $31.1 billion, or 76.4% of the world’s wine exports, Europe had the largest dollar export value of any continent in 2021. Australia and New Zealand led Oceania’s sales of imported wine, which were 7.5% higher than Latin America’s 7.1%, which included the Caribbean but excluded Mexico^2,3^. North American wine exporters provided 3.8% of the world’s wine exports, while Asia delivered 3.3% ahead of 1.9% of sales of wine from African producers.

In summary, the wine industry is investing in technology to improve both sales and production of wine, and quality evaluation and certification are essential factors in this process. Certification helps protect human health by preventing unlawful wine adulteration and ensuring market quality. The certification process^4^ often involves evaluating wine quality using physicochemical and sensory tests, with physicochemical tests being based on laboratory measurements and sensory tests relying on human expertise. However, the relationship between physicochemical and sensory analysis is complex and not fully understood, making it challenging to classify wine accurately based on taste.

The advancement of information technology has made it possible to collect, store, and analyse large, complex datasets to improve decision-making and increase the likelihood of success. Machine learning algorithms are used to create sophisticated knowledge from unstructured data. Various machine learning algorithms^5^ include linear and multivariate regression, neural networks, and support vector machines. These algorithms have different benefits and are helpful for different types of data. However, it is essential to carefully select the appropriate variables and models when using these methods. A too-simple model may not effectively convey the underlying idea, and a too-complex model may oversample the data.

In this research, we show a practical application in which multiple machine-learning models are used to simulate wine taste preferences using readily accessible analytical data at the certification stage. Unlike past investigations, a sizable dataset with 1599 samples is considered. Explanatory information is provided through a sensitivity analysis, which evaluates how the answers are affected when the input domain is modified. The sensitivity analysis serves as a reference for the simultaneous selection of the variable and the model. In order to get the optimum parameters with the least amount of computational work, we also examine five different machine learning models: Random Forests (RF), Decision Trees (DT), AdaBoost, Gradient Boost, and Extreme Gradient Boosting (XGBoost)^6,7^.

A potent ensemble learning approach called Random Forests (RF) combines several decision trees to improve prediction accuracy and decrease overfitting. Each decision tree in the forest is built using a different random subset of data and characteristics. RF combines the output of each individual tree during prediction, frequently by majority vote for classification and average for regression. Fundamental to machine learning are decision trees (DT), which depict a tree-like structure with core nodes denoting features, branches denoting decision rules, and leaf nodes yielding outcomes. DTs create a decision tree iteratively by segmenting data according to useful attributes. From the base of the tree to the leaf, when the final forecast is produced, decisions are taken.

An ensemble approach called AdaBoost (Adaptive Boosting) combines weak learners, often decision trees, into a powerful model. It weights the data points and develops a number of ineffective classifiers. AdaBoost refines the model by emphasising misclassified data points repeatedly; the final prediction is a weighted composite of weak learners. Another ensemble method, gradient boosting, builds an additive model by successively adding weak learners. It is helpful for classification and regression applications because it reduces the gradient of a loss function. It starts with an initial prediction and iteratively fits weak learners to forecast residual errors, eventually improving the predictions of the model.

An effective kind of gradient boosting recognised for its great performance is called extreme gradient boosting (XGBoost). To improve forecast accuracy, it uses parallel processing, regularisation, and tree pruning. To avoid overfitting, XGBoost builds decision trees sequentially and adds regularisation terms. To maximise model performance, it makes use of strategies like feature significance and early stopping. The unique dataset and issue features will determine which model is used, and each model has strengths and drawbacks of its own. In contrast to boosting techniques like AdaBoost and XGBoost, which may provide excellent accuracy with careful tuning, Random Forests are resilient and resistant to overfitting. Decision trees can be understood, but they are prone to overfitting.

Finally, we show how established models have impacted the wine domain. To assess the precision of its predictions, we also trained, calibrated, and tweaked an RF classifier's hyperparameters. We have also undertaken cluster analysis to manage collinearity and limit the number of predictors without compromising model accuracy. The objectives of Wine Quality Modelling and Prediction Using Various Machine Learning Models are as follow:

- Wine quality modeling using its physicochemical characteristics.
- To test various ML-based classification techniques to determine which delivers the highest accuracy.
- To identify the characteristics of a high-quality wine that are most significant.
- To manage collinearity and cut down on predictors without compromising model accuracy.

The rest of the work is organized as follows. Section “Related work” presents the related work. Section “Materials and methods” presents the material and methods employed in wine quality modeling and prediction, the RWD dataset, data Pre-processing & exploratory data analysis, and ML Analysis and feature selection. The hyperparameter tuning and clustering analysis, along with the discussions, is illustrated in section “Hyperparameter tuning and clustering analysis”. This section discusses the key conclusions of pertinent papers, and the literature supports noteworthy discoveries from the ongoing study. The conclusion and future recommendations are highlighted in the final section.

## Related work

The wine business mainly uses ML techniques during the wine production process. Despite the ability of ML models to forecast wine quality based on physicochemical data, their use is quite limited and often considers small datasets. This section presents the literature review of the very popular and highly cited work.

In several crucial ways, the authors’ research sets itself apart from earlier investigations. First off, their use of the red wine dataset with its eleven unique physiochemical traits makes it unique and may provide insights into a dataset for wine quality forecasting that hasn’t been well studied. Second, their inventive feature selection strategy may offer a special way to find the most important characteristics. It makes use of machine learning methods like Random Forest and Extreme Gradient Boosting. The authors also want to demonstrate greater model performance, maybe by careful model calibration and hyperparameter adjustment. Their research differs from others in terms of technique due to the use of clustering techniques for data preparation. Finally, their research goals or applications connected to wine quality prediction may provide fresh perspectives or useful uses that haven’t been widely explored in earlier papers. These disparities add up to the structural and theoretical divergences between the authors’ study and the body of prior work in the topic.

Using objective hypothesis testing accessible at the certification stage, Cortez et al.^8^ aim to anticipate wine tastes. White Vinho Verde samples from northwest Portugal were included in a significant dataset. Regression analysis was used to analyse this case study. The regression model modelled wine preference on a continuous scale from 0 to 10. An efficient and robust process that simultaneously selects variables and modelling and is directed by sensitivity analysis was used to apply three regression techniques. Agarwal et al.^9^ evaluate how a deep learning algorithm forecasts for quality by employing two different convolution layers rather than focusing on various approaches. It will let winemakers use deep learning to judge how to manage their operations. The experiment's limited data set and feature set made it impossible for a machine to choose the most helpful characteristics. By considering several feature selection techniques, such as the Recursive Feature Elimination method (RFE) and Principal Component Measurement (PCA) for feature selection, as well as non-linear decision tree-based classifiers for the analysis of performance indicators, Aich et al.^10^ developed a new technique. Their investigation can aid wine specialists in understanding the crucial elements to consider when choosing high-quality wines.

Gupta et al.’s^11^ machine learning algorithm with a user interface forecasts the wine quality by selecting the key wine factor vital for determining the wine quality. The Random Forest method evaluates wine quality, and KNN is used to improve the model’s accuracy further. The result of the suggested model is utilized to assign the wines a Good, Average, or Bad quality rating. The goal of Kumar et al. study^12^ is to determine the quality of red wine using a range of its characteristics. Methods like RF, SVM, and NB are employed, and the dataset is gathered from the sources. The outcomes are compared between the training dataset and testing set, several performance measures are computed, and the optimum of the three techniques is therefore predicted based on the learning set outcomes. Shaw et al.^13^ compares the SVM, RF, and multilayer perceptron classification algorithms for wine quality analysis to determine which algorithm produces the most accurate results. The multilayer perceptron algorithm comes in second place with an accuracy of 78.78%, followed by the SVM algorithm with an accuracy of 57.29% during our comparative analysis between those algorithms. The RF algorithm produces the best results with an accuracy of 81.96%.

Bhardwaj et al.^14^ examined the chemical and physicochemical data from New Zealand Pinot Noir wines. The 18 samples contained 54 characteristics. The remaining 47 qualities are linked to chemical data, while 7 of the 54 characteristics are tied to physiochemical data. Four different feature selection techniques were used to compare their findings. Significant attributes that were proven to be useful in at least three feature selection methods were used to predict wine quality. On an original holdout sample, seven machine learning algorithms were trained and put to the test. Tiwari et al.^15^ employ a mathematical model that makes use of metrics for perceived wine quality by the industry professionals and wine specialists studied. The relevant sensory and chemical concepts are then validated using ML methods. Two sets of 18 New Zealand Pinot Noir wines were evaluated by wine experts on their inherent qualities, including overall quality. To predict wine quality, they develop a conceptual and mathematical framework. It then uses machine learning techniques to test these frameworks using a huge dataset.

Table 1 below compares the literature review of popular and highly cited work in this domain.

**Table 1:** Comparison of literature review of the very popular and highly cited work.

Study	ML technique	Wine characteristics considered	Prediction of wine quality	Results	Drawbacks
Cortez et al.^8^	Regression	Physicochemical data	Continuous scale from 0 to 10	High prediction accuracy	N/A
Agarwal et al.^9^	Deep learning	Limited data set and features	N/A	N/A	Limited data set and available features
Aich et al.^10^	PCA, RFE, Nonlinear decision tree-based classifiers	N/A	Crucial elements for choosing high-quality wines	N/A	N/A
Mahima et al.^16^	Random Forest, KNN	N/A	Good, Average, or Bad	Improved prediction accuracy with KNN	N/A
Kumar et al.^12^	RF, SVM, NB	N/A	N/A	Comparison of performance metrics between training and testing sets	N/A
Shaw et al.^13^	SVM, RF, Multilayer perceptron	N/A	Accuracy comparison	RF produces best results with accuracy of 81.96%	N/A
Bhardwaj et al.^14^	Four feature selection techniques, seven machine learning algorithms	Chemical and physicochemical data	Significant attributes for predicting wine quality	N/A	N/A
Tiwari et al.^15^	Mathematical model, ML techniques	Sensory and chemical data	Wine quality	N/A	N/A
Ma et al.^17^	Deep learning	Physicochemical data	Wine type	High prediction accuracy	N/A
Prez et al.^18^	PCA, SVM	Physicochemical data	Wine quality	N/A	N/A
Gupta et al.^11^	RF, SVM, NB	Sensory data	Wine quality	N/A	N/A

## Material and methods

This study’s analysis was completed using the Google Colab notebook, Python version 3.8.16. The operating system that was installed on the system was Windows 10 64-bit. An NVIDIA GeForce 1 GB graphics card and an Intel i5-Core 2.5 GHz processor with 8 GB RAM round out the hardware specifications. This work is implemented by programming with Python, proposing the framework, and using the classifier provided by the Scikit-Learn package.

### Datasets

There are two datasets regarding the red and white varieties of Portuguese “Vinho Verde” wine. This project uses only the red wine dataset (RWD)^15^. Paulo Cortez’s website and the UCI Machine Learning Repository have available datasets. This study examined the physicochemical variables as input and sensory variables as output from available due to logistical and privacy concerns (for instance, there is no data about grape types, wine brands, wine selling price, etc.). There are far more average wines than good or bad ones, and the classes are organized but unbalanced.

**Figure 2:**
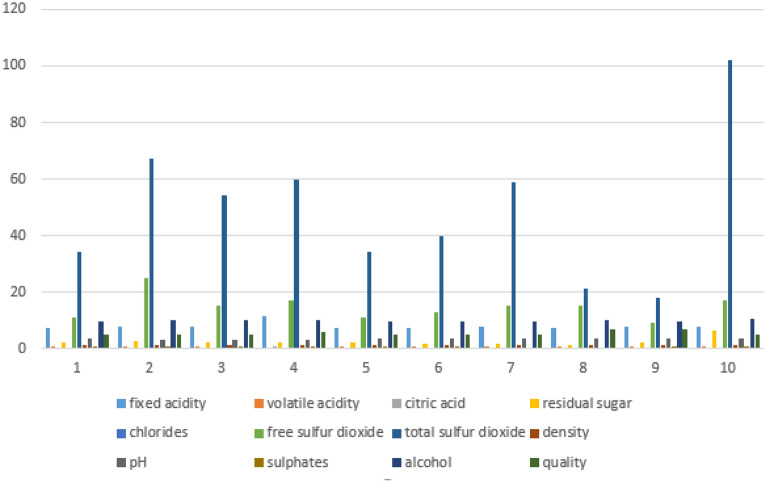
Snapshot of the RWD.

**Figure 3:**
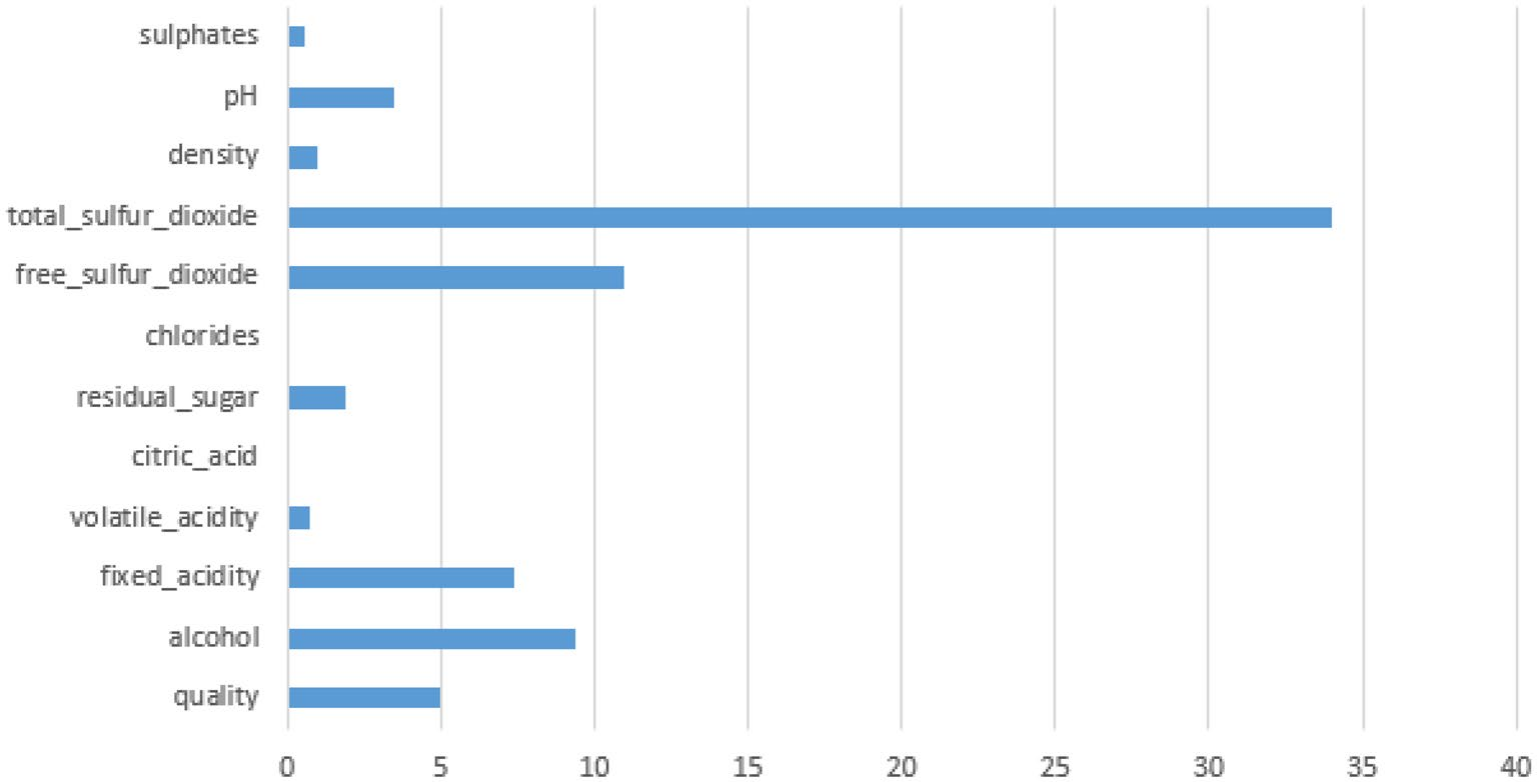
Plot of physicochemical attributes of RWD.

There were 11 features in the RWD (Alcohol, Chlorides, Citric Acid, Density, Fixed Acidity, Free Sulphur Dioxide, Ph, Residual Sugar, Sulphates, Total Sulphur Dioxide, and Volatile Acidity). The Categorical levels from 3 to 8. The quality of the wine is used to perform classification tasks. Data samples from Red Wine Quality Dataset mentioned in Table 2. Figure 2 displays the snapshot of the RWD and Fig. 3 illustrates the plot for the physicochemical characteristics of RWD. Sulphates, chlorides, pH, alcohol, quality, density, and other physicochemical properties are examples. Here, the RWD has a pH of 3.51, which is very acidic, and a density of 99.78%. Table 2 displays the data samples from red wine.

**Table 2:** Data samples from red wine quality dataset.

Sr. no	Fixed acidity	Volatile acidity	Citric acid	Residual sugar	Chlorides	Free sulfur dioxide	Total sulfur dioxide	Density	Ph	Sulphates	Alcohol	Quality
1	6.6	0.725	0.2	7.8	0.073	29.0	79.0	0.9977	3.29	0.54	9.2	5
2	6.3	0.55	0.15	1.8	0.077	26.0	35.0	0.99314	3.32	0.82	11.6	6
3	5.4	0.74	0.09	1.7	0.089	16.0	26.0	0.99402	3.67	0.56	11.6	6
4	6.3	0.51	0.13	2.3	0.076	29.0	40.0	0.99574	3.42	0.75	11.0	6
5	6.8	0.62	0.08	1.9	0.068	28.0	38.0	0.99651	3.42	0.82	9.5	6
6	6.2	0.6	0.08	2.0	0.09	32.0	44.0	0.9949	3.45	0.58	10.5	5
7	5.9	0.55	0.1	2.2	0.062	39.0	51.0	0.99512	3.52	0.76	11.2	6
8	6.3	0.51	0.13	2.3	0.076	29.0	40.0	0.99574	3.42	0.75	11.0	6
9	5.9	0.645	0.12	2.0	0.075	32.0	44.0	0.99547	3.57	0.71	10.2	5
10	6.0	0.31	0.47	3.6	0.067	18.0	42.0	0.99549	3.39	0.66	11.0	6

### Data pre-processing & exploratory data analysis

**Figure 4:**
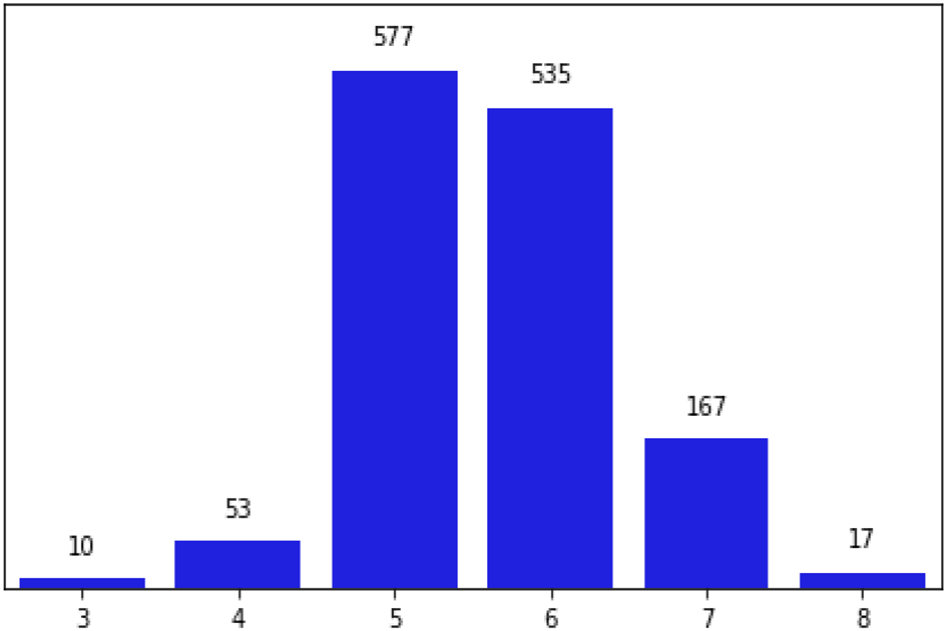
Plot for wine quality.

**Figure 5:**
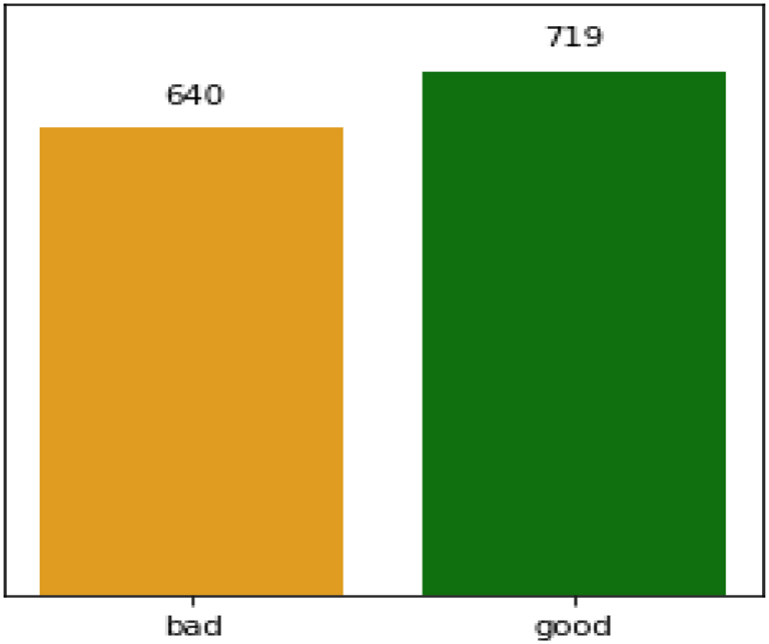
Comparison plot for two classes of RWD.

The RWD has total of 1599 rows and 12 columns in which one of the columns shows the quality of the wine evaluated with discrete values between 3 and 8 as illustrated in Fig. 4. The rest of the columns correspond to the physicochemical attributes like fixed acidity, volatile acidity, citric acid, residual sugar, chlorides, free sulphur dioxide, total sulphur dioxide, density, pH, sulfates, and alcohol. We have converted the output to a binary classification problem where each wine is either “good quality” or not. The comparison plot for two classes of wine quality is shown in Fig. 5. For many ML models, a resampling technique like K-fold Cross-Validation Synthetic Minority Oversampling Technique (SMOTE) may be required if the data was extremely imbalanced, but in this case, the data seems to be balanced as 640 being classified in bad quality and 719 are classified as good quality. First, the duplicate values present in the dataset is dropped to perform data cleaning. After that, the index is reset to make the data uniform. We normalized the data by changing it so that its distribution will have a mean of 0 and a standard deviation of 1 to prepare it for modelling. Standardizing the data is crucial to equating the data's range and preventing bias. The ML models were trained using this dataset.

### Machine learning analysis

**Figure 6:**
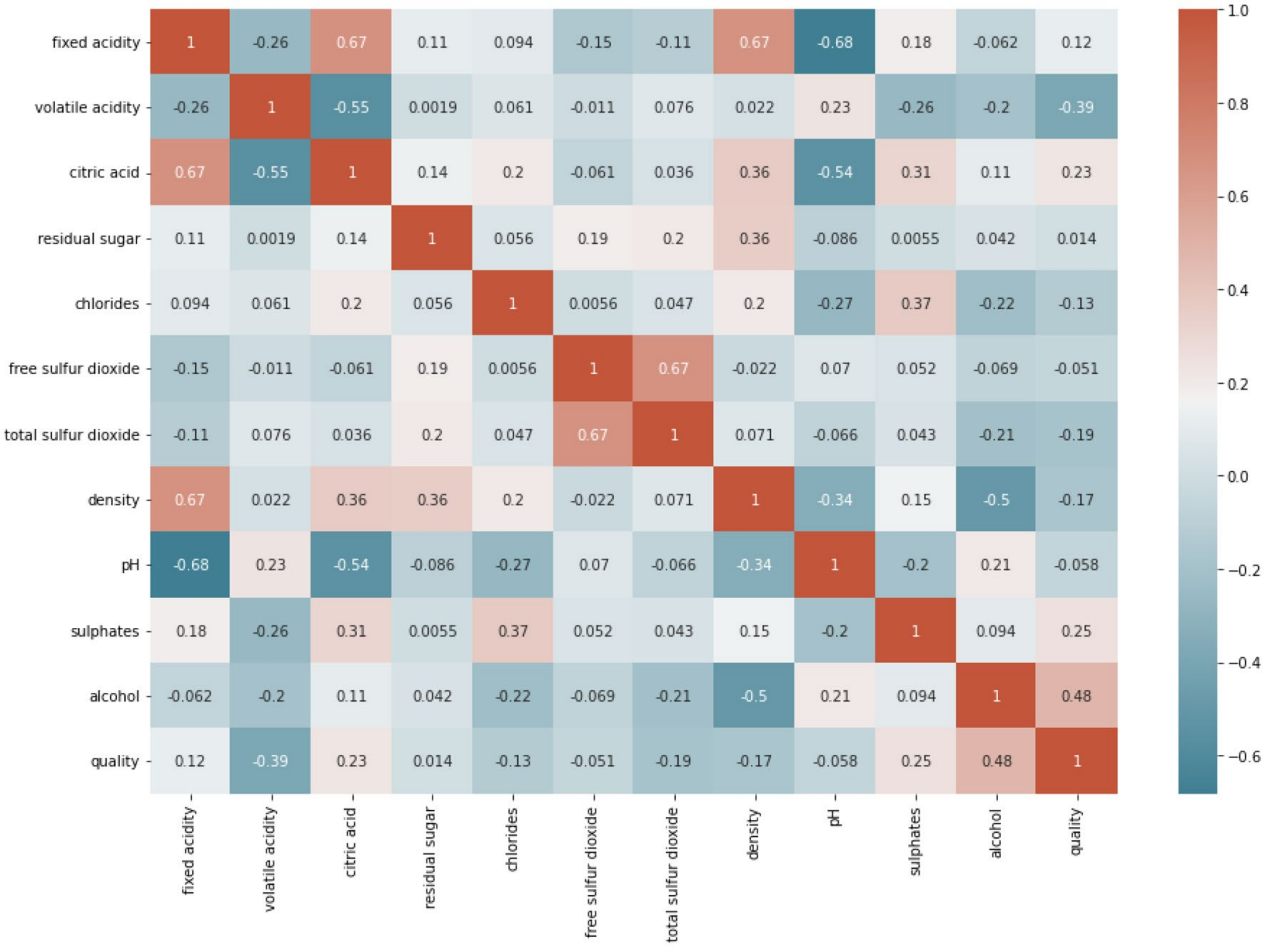
Correlation matrix for the features of RWD.

To establish the correlation between the various features to understand their relationships better, we plot a correlation plot between all the features of RWD, as represented in Fig. 6. As seen from this figure that some features that are strongly correlated to *quality.* So, these variables are also the most important features in the ML Analysis and models. To compare the performance of various classifiers, we have already transformed the output variable to a binary as “good quality” if $quality\ge 7$ and ‘bad quality’ if if $quality<7$. The feature variables $(X)$ will also be separated from the target variable $(Y)$ into different data frames. To cross-validate the ML models and assess their efficacy, we have divided the data into training and test sets at 80% and 20%.

We have compared five different machine learning models, DT, RF, AdaBoost, Gradient Boost, and XGBoost, for their accuracy. An ensemble machine learning approach called gradient boosting is well known for its exceptional predictive power in regression and classification applications. This technique works by sequentially assembling a group of weak learners, frequently decision trees. It begins with an initial forecasting, usually a straightforward one, and then moves on to find and fit additional weak learners who are precisely targeted at the residuals or mistakes generated by the current ensemble. With each iteration, these fresh recruits are carefully chosen to cut down on mistakes and eventually increase the model's accuracy. By merging the predictions from each weak learner, the final prediction is made, creating a powerful and incredibly accurate predictive model. In operations research, strategic planning, and ML, decision trees are a common model. Although they are simple to construct and intuitive, decision trees are inaccurate. An ensemble learning method based on decision trees is called random forests. Several decision trees are constructed using random forests utilizing bootstrapped datasets of the original information, which then randomly select a subset of the variables for each step of the decision tree. The model then selects the mode of each decision tree's predictions. By relying on a “majority wins” approach, the likelihood of a single tree making a mistake is reduced.

The following three models use boosting techniques to make weak learners stronger. Both regression and classification problems can be addressed with gradient-boosting techniques. Although they are generally used with tree-based models, they could theoretically be used with any weak learner. This study’s^19^ objective is to evaluate the accuracy of subjective quality measurements for Italian wines (Barolo and Barbaresco) dependent on the seasonal weather. The study used an ordered probit model to evaluate the factors that affect vintage quality ratings and to shed light on the validity of expert assessments. The findings of this study may help decipher the variables that affect wine quality and determine the validity of professional assessments of wine quality.

This work^20^ is the first to estimate the alcoholic content, sugar content, and overall acidity of straw wine using mid-infrared (MIR) spectroscopy and multivariate data processing. To build novel regression functions based on a mix of orthogonal signal correction and partial least squares regression to predict quality characteristics, the study assessed 302 Italian wine samples using MIR spectroscopy and reference techniques. With this strategy, we want to minimize matrix complexity, lessen spectrum interference, and enhance the information in fingerprinting data. The findings of this study may help understand the variables that affect wine quality and create more precise methods for wine quality prediction.

This study^21^ used a combination of physical–chemical analysis to monitor the aging process of red wines from D.O. Toro (Spain) and distinguish between wine samples aged for different periods. Different computational models, including artificial neural network models (ANNs), support vector machines (SVMs), and random forest (RF) models, were developed to serve as an authenticity tool for certifying wines. The results of this study may be useful for understanding the changes in the chemical composition of wines over time and for developing tools to certify the authenticity of wines. Gradient boosting attempts to explain the patterns that the preceding weak learner missed by sequentially fitting weak learners to the gradient (derivative) of a loss function. The weak learners are combined using an additive model as each one is fitted^22^. To modify the predictions, the output of the new weak learner is added to the output of the prior weak learner. As a result, an equation becomes recursive, with each poor learner attempting to explain a pattern that the previous ones had missed. A constant, like the mean, is used to initialize the first weak learner.

1 $$y\approx {F}_{0}\left(x\right)=\frac{i}{n}\sum_{i=0}^{M}{y}_{i}.$$

The residuals are then fit with a function called $h(x)$. The gradient of the loss function can be found in the residuals.

2 $${F}_{1}\left(x\right)={F}_{0}\left(x\right)+ {h}_{0}\left(x\right),$$

3 $${F}_{2}\left(x\right)={F}_{1}\left(x\right)+ {h}_{1}\left(x\right),$$

$$\cdots$$

4 $${F}_{m+1}\left(x\right)={F}_{m}\left(x\right)+ {h}_{m}\left(x\right),$$

where $h(x)$ is a weak learner fit to a loss function's gradient. $\Upsilon$ stands for the step size or learning rate.

5 $${h}_{m}\left(x\right)=\Upsilon \nabla L\left(y,{F}_{m}\left(x\right)\right).$$

Each feature in the final model is represented by a number of terms, each of which influences the prediction in a different way.

6 $$y\approx \widehat{{F}_{M}\left(x\right)}=\left(\sum_{m=1}^{M}{h}_{m}\left(x\right)\right)+ {F}_{0}\left(x\right).$$

This method can be used to solve classification and regression issues since any differentiable loss function can be chosen because the weak learners are fit to predict the gradient of the loss function. Gradient Boosting has been improved upon with Extreme Gradient Boosting (XGBoost). The model generalization abilities of XGBoost are enhanced by the application of advanced regularization (L1 & L2). When compared to gradient boosting, XGBoost offers exceptional performance. Its parallelization across clusters and quick training speed. When a model is being evaluated, a classification report will include the precision, recall, and F1 score. These results may be used to evaluate the effectiveness of the whole ML model discussed above. The Output of various ML models are presented in Table 3.

**Table 3:** Output of various ML techniques.

Machine learning models	Class	Precision	Recall	F1-score	Support
Decision tree	0	0.97	0.92	0.94	290
	1	0.49	0.77	0.60	30
	Accuracy			0.90	320
	Macro avg	0.73	0.84	0.77	320
	Weighted avg	0.93	0.90	0.91	320
Random forest	0	0.96	0.97	0.96	290
	1	0.65	0.57	0.61	30
	Accuracy			0.93	320
	Macro avg	0.80	0.77	0.78	320
	Weighted avg	0.93	0.93	0.93	320
AdaBoost	0	0.94	0.96	0.95	290
	1	0.52	0.43	0.47	30
	Accuracy			0.91	320
	Macro avg	0.73	0.70	0.71	320
	Weighted avg	0.90	0.91	0.91	320
Gradient boosting	0	0.94	0.94	0.94	290
	1	0.52	0.51	0.52	30
	Accuracy			0.89	320
	Macro avg	0.73	0.73	0.73	320
	Weighted avg	0.89	0.89	0.89	320
XG boosting	0	0.94	0.95	0.95	290
	1	0.57	056	0.56	30
	Accuracy			0.90	320
	Macro avg	0.76	0.75	0.75	320
	Weighted avg	0.90	0.90	0.90	320

When evaluating the five models, it appears that the RD and XGBoost produce the most accurate results. However, RF is more accurate at predicting high-quality wines thanks to its higher f1-score. Therefore, further experiments we have selected for performing hyperparameter tuning and implementing clustering analysis.

### Feature selection

Induction algorithms occasionally become inefficient and, if not fully utilised, can waste a significant amount of memory and/or time due to their vast number of input parameters. Furthermore, irrelevant data may confuse algorithms, leading them to make faulty conclusions and deliver subpar results. Improved comprehension and less expensive data collection and handling costs are two advantages of feature selection. Due to these advantages, feature selection received much attention from the ML and Data Mining sectors. Several techniques have been developed, but some of the most well-known feature selection algorithms include the XGB and RF classifiers.

Different methods are used by machine learning models to choose the optimal characteristics for a job. Feature significance analysis, a popular technique, evaluates the contribution of each feature to the model's predictive capability. A feature's significance score is calculated by some models, such as Decision Trees and Random Forests, depending on how frequently a feature is used to divide the data and the impurity reduction it provides. Another method is known as recursive feature elimination (RFE), which starts with all characteristics and gradually eliminates the ones that aren’t crucial based on how well a model performs. Additionally, models could automatically decrease coefficients to zero using strategies like L1 regularisation (Lasso), which successfully selects pertinent characteristics. To identify the subset of characteristics that best optimises a model’s accuracy and generalisation, feature selection may also entail domain expertise and experimentation. To improve model performance and interpretability, the feature selection approach is chosen based on the dataset, the particular model, and the task at hand.

**Figure 7:**
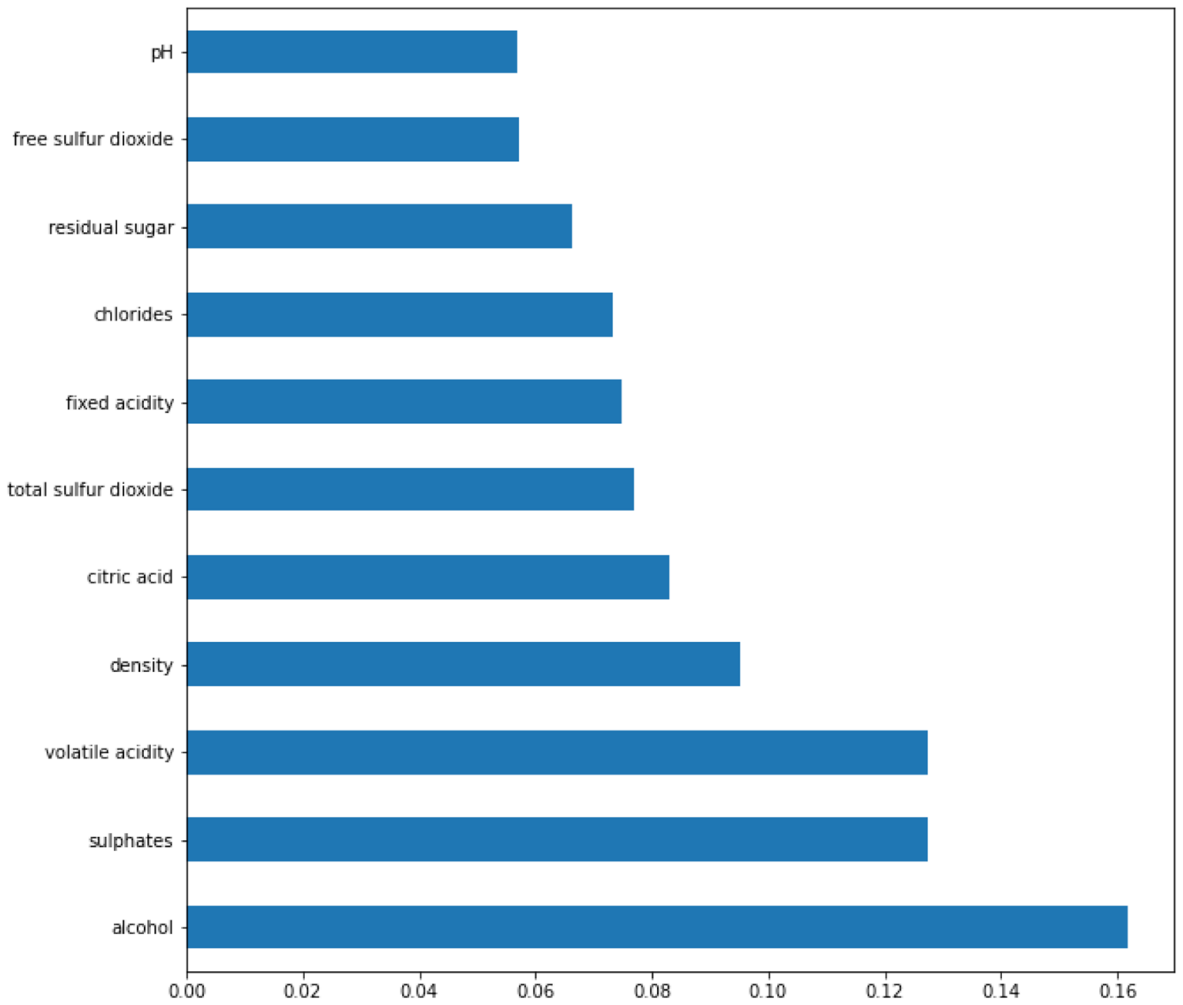
Feature importance based on the RF model. The top four features are alcohol, sulphates, volatile acidity and density.

**Figure 8:**
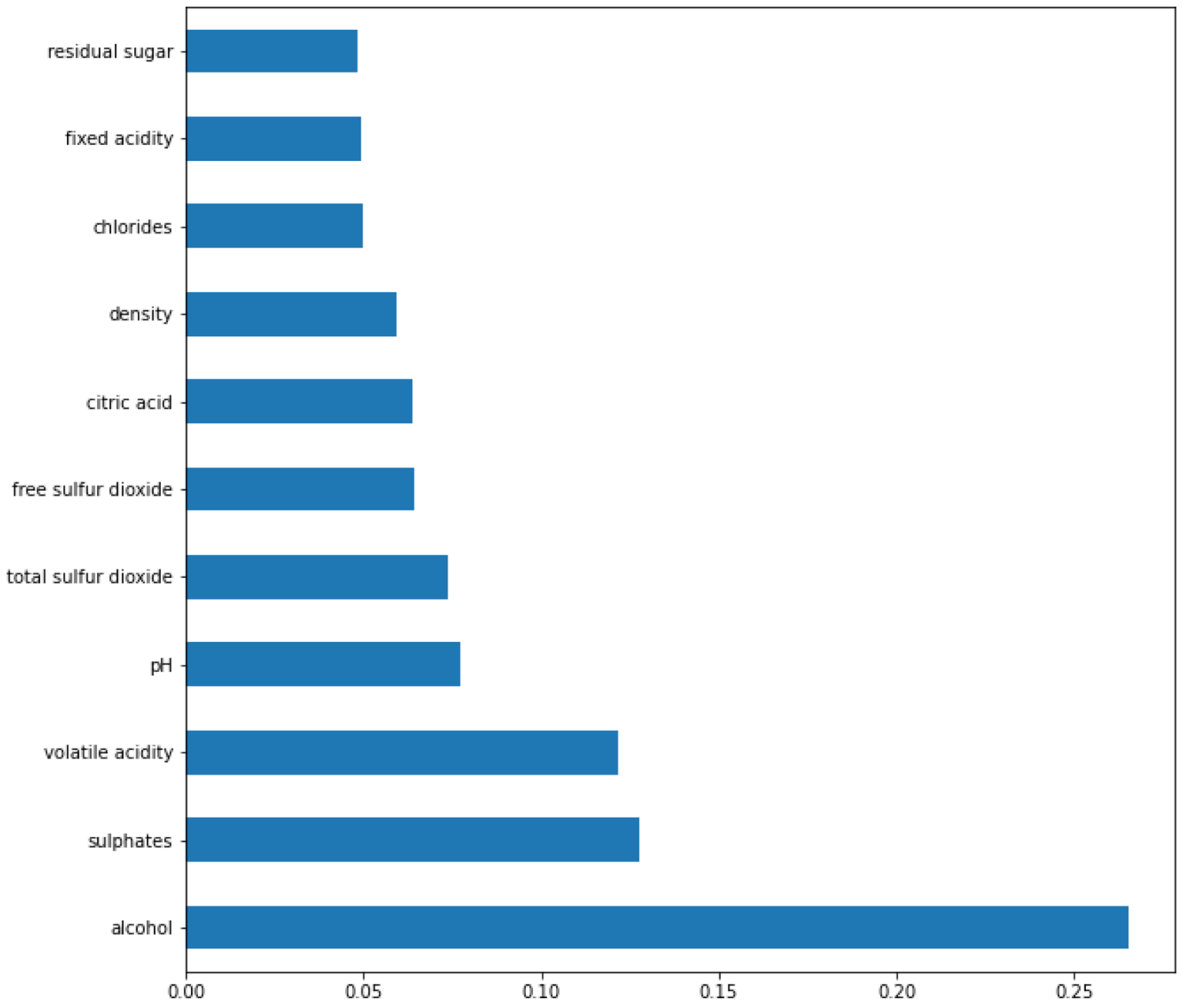
Feature importance based on the XGBoost model. The top four features are: alcohol, sulphates, volatile acidity and pH.

In this section, we used these two ML methods to extract the best three features from eleven features, and we then executed ML analysis on the returned features. The quality of the wine was estimated using ML techniques. As already stated, the RWD is categorized as a binary classification problem. The default parameters for each ML classifier were utilized. We graphed the feature importance based on the RF model in Fig. 7 and the XGBoost model in Fig. 8. While they vary slightly, the top 3 features are the same: alcohol, sulfates, and volatile acidity.

By examining these descriptive statistics from Tables 4 and 5, we can infer that superior-quality wines typically include higher amounts of alcohol, lower volatile acidity, higher levels of sulfates, and higher levels of residual sugar.

**Table 4:** Descriptive statistics of “good quality” wine.

Index	Fixed acidity	Volatile acidity	Citric acid	Residual sugar	Chlorides	Free sulfur dioxide	Total sulfur dioxide	Density	Ph	Sulphates	Alcohol	Quality	Good quality
Count	217.0	217.0	217.0	217.0	217.0	217.0	217.0	217.0	217.0	217.0	217.0	217.0	217.0
Mean	8.8470	0.4055	0.3764	2.7087	0.07591	13.9815	34.8894	0.9960	3.2888	0.7434	11.5180	7.0829	1.0
Std	1.9999	0.1449	0.1944	1.3630	0.02848	10.2346	32.5722	0.0022	0.1544	0.1340	0.9981	0.2764	0.0
Min	4.9	0.12	0.0	1.2	0.012	3.0	7.0	0.9906	2.88	0.39	9.2	7.0	1.0
25%	7.4	0.3	0.3	2.0	0.062	6.0	17.0	0.9947	3.2	0.65	10.8	7.0	1.0
50%	8.7	0.37	0.4	2.3	0.073	11.0	27.0	0.9957	3.27	0.74	11.6	7.0	1.0
75%	10.1	0.49	0.49	2.7	0.085	18.0	43.0	0.9973	3.38	0.82	12.2	7.0	1.0
Max	15.6	0.915	0.76	8.9	0.358	54.0	289.0	1.0032	3.78	1.36	14.0	8.0	1.0

**Table 5:** Descriptive statistics of “bad quality” win.

Index	Fixed acidity	Volatile acidity	Citric acid	Residual sugar	Chlorides	Free sulfur dioxide	Total sulfur dioxide	Density	Ph	Sulphates	Alcohol	Quality	Good quality
Count	1382.0	1382.0	1382.0	1382.0	1382.0	1382.0	1382.0	1382.0	1382.0	1382.0	1382.0	1382.0	1382.0
Mean	8.2368	0.5470	0.2544	2.5121	0.08928	16.1722	48.2858	0.9968	3.3146	0.6447	10.2510	5.4088	0.0
Std	1.6827	0.1763	0.1896	1.4157	0.04911	10.4676	32.5856	0.0018	0.1541	0.1706	0.9696	0.6017	0.0
Min	4.6	0.16	0.0	0.9	0.034	1.0	6.0	0.990	2.74	0.33	8.4	3.0	0.0
25%	7.1	0.42	0.0825	1.9	0.071	8.0	23.0	0.9957	3.21	0.54	9.5	5.0	0.0
50%	7.8	0.54	0.24	2.2	0.08	14.0	39.5	0.9968	3.31	0.6	10.0	5.0	0.0
75%	9.1	0.65	0.4	2.6	0.091	22.0	65.0	0.9979	3.41	0.7	10.9	6.0	0.0
Max	15.9	1.58	1.0	15.5	0.611	72.0	165.0	1.0036	4.01	2.0	14.9	6.0	0.0

## Hyperparameter tuning and clustering analysis

As discussed in section “Machine learning analysis”, the RD and XGBoost yield the most accurate results out of the five models. However, RF is more accurate at predicting high-quality wines thanks to its higher f1-score. Therefore, we have selected RF for performing hyperparameter tuning and implementing clustering analysis for further experiments. In order to find the best configuration of hyperparameters for a particular problem, one must first choose the hyperparameters for a machine learning model, define a search space with specific ranges or values for each hyperparameter (for example, learning rate, depth of trees), and then use search techniques like grid search or random search. The F1 score is a statistic that balances accuracy and recall by combining both precision and recall into a single value. It is especially helpful for evaluating a model’s effectiveness while dealing with unbalanced datasets. The fraction of accurate positive predictions among all positive predictions generated by the model is measured by precision, also known as Positive Predictive Value. It serves as a gauge of how accurately the good forecasts were made. Recall, sometimes referred to as Sensitivity or True Positive Rate, calculates the percentage of true positive forecasts among all occurrences of positive data that occurred. It is a gauge of how well the model captures all occurrences of positivity.

### Performing hyperparameter tuning

**Figure 9:**
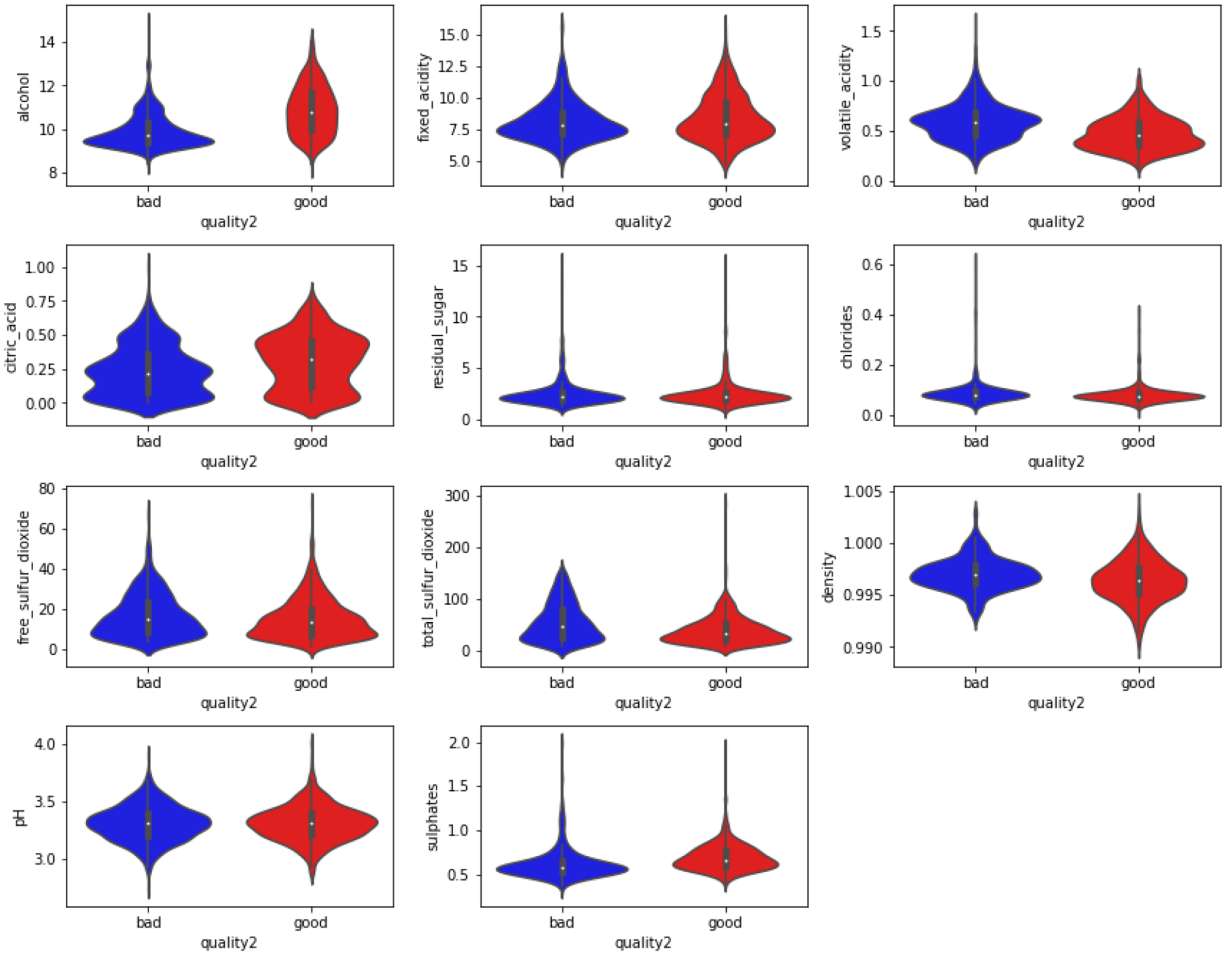
Violin plot for all the eleven features of the WDS with “good quality” and “bad quality wine.

We further implemented the violin plot in Fig. 9 for all the eleven features of the WDS with “good quality” and “bad quality wine, which depicts data peaks, is a combination between a box plot and a kernel density plot. It is used to show how numerical data is distributed and summary statistics as well as the density of each feature of the dataset, unlike box plots, which can only show summary statistics. Thus, violin plots and box plots share many of the same summary statistics, apart from the points that are designated as “outliers” using a method that is a function of the interquartile range. The interquartile range (IQR) is shown by the thick black bar in the middle, the median is shown by the white dot, and the remainder of the distribution is shown by the thin grey line. To display the data's distribution shape, a kernel density estimation is placed on either side of the grey line. A larger probability that the provided value will be adopted by population members is shown by wider areas of the violin plot, whilst a lesser probability is represented by skinnier sections.

**Figure 10:**
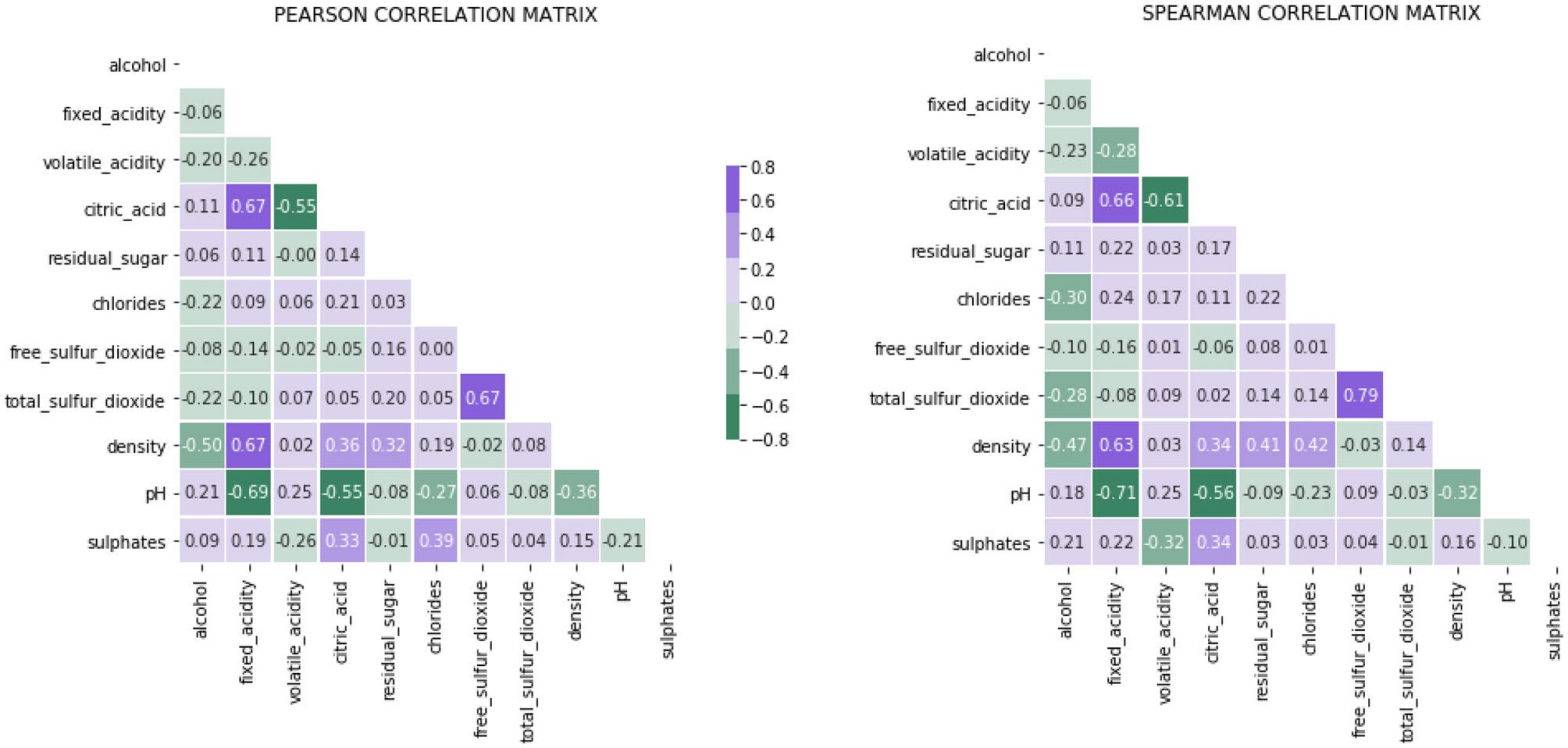
Pearson corelation matrix and spearman corelation matrix.

A correlation matrix is represented as a correlogram. Highlighting the features of a dataset that are highly connected is quite helpful. According to their values, correlation coefficients are coloured in this graph. The correlation matrix can also be rearranged in accordance with how strongly two variables are related. Figure 10 display the Pearson corelation matrix and spearman corelation matrix.

**Figure 11:**
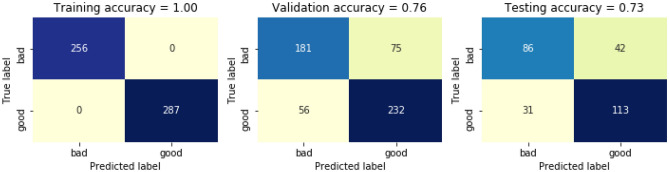
Confusion matric after applying RF for the above distribution of classes (Table 6).

After defining feature dataset and encoding the class labels, we generate training sets, validation sets and testing sets for the WDS. Total number of rows in the datasets are 1359. Out if which 543 for training, 544 for validation and 272 for testing. The distribution by the class is further represented in Table 6. The data visualizer is presented in the form of confusion matric after applying RF for the above distribution of classes in Fig. 11. Here the training accuracy is 100%, whereas the validation accuracy and testing accuracy is 76% and 73% respectively, thus giving a total accuracy of 85%.

**Table 6:** Distribution by classes.

Class	Training set	Validation set	Test set
0	256	256	128
1	287	288	144

**Figure 12:**
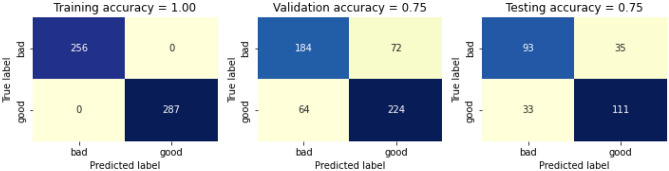
Confusion matric after calibrating the RF model.

Model calibration is the process of selecting a set of model parameters that best captures the behaviour of the system. It is accomplished by contrasting model predictions with actual system data. Thus, we have computed the confusion matrix after calibrating the above model. The data visualizer is presented in the form of confusion matric after applying calibrating the model in Fig. 12. Here the training accuracy is 100%, whereas the validation accuracy and testing accuracy is 75% and 75% respectively, thus giving a total accuracy of 85%.

**Figure 13:**
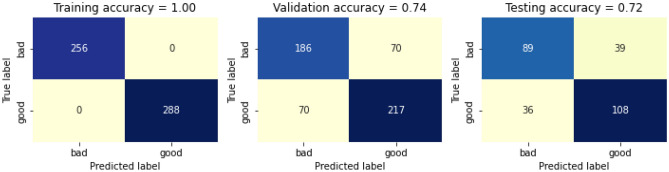
Confusion matricbefore calibrating after swapping the roles between training dataset and validation datasets.

**Figure 14:**
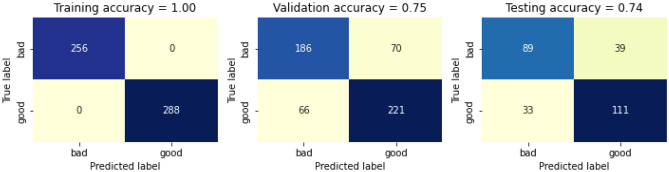
Confusion matricafter calibrating after swapping the roles between training dataset and validation datasets.

We further evaluated the performance of the model by swapping the roles between training dataset and validation datasets. The confusion matrix before calibrating the swapped dataset is presented in Fig. 13. The training accuracy before calibrating the swapped dataset is 100%, whereas the validation accuracy before calibrating is 74% and 72% respectively, thus giving a total accuracy before calibrating is 84%. The confusion matrix after calibrating the swapped dataset is presented in Fig. 14. The training accuracy after calibrating the swapped dataset is 100%, whereas the validation accuracy and testing accuracy before calibrating is 75% and 74% respectively, thus giving a total accuracy before calibrating is 85%.

**Figure 15:**
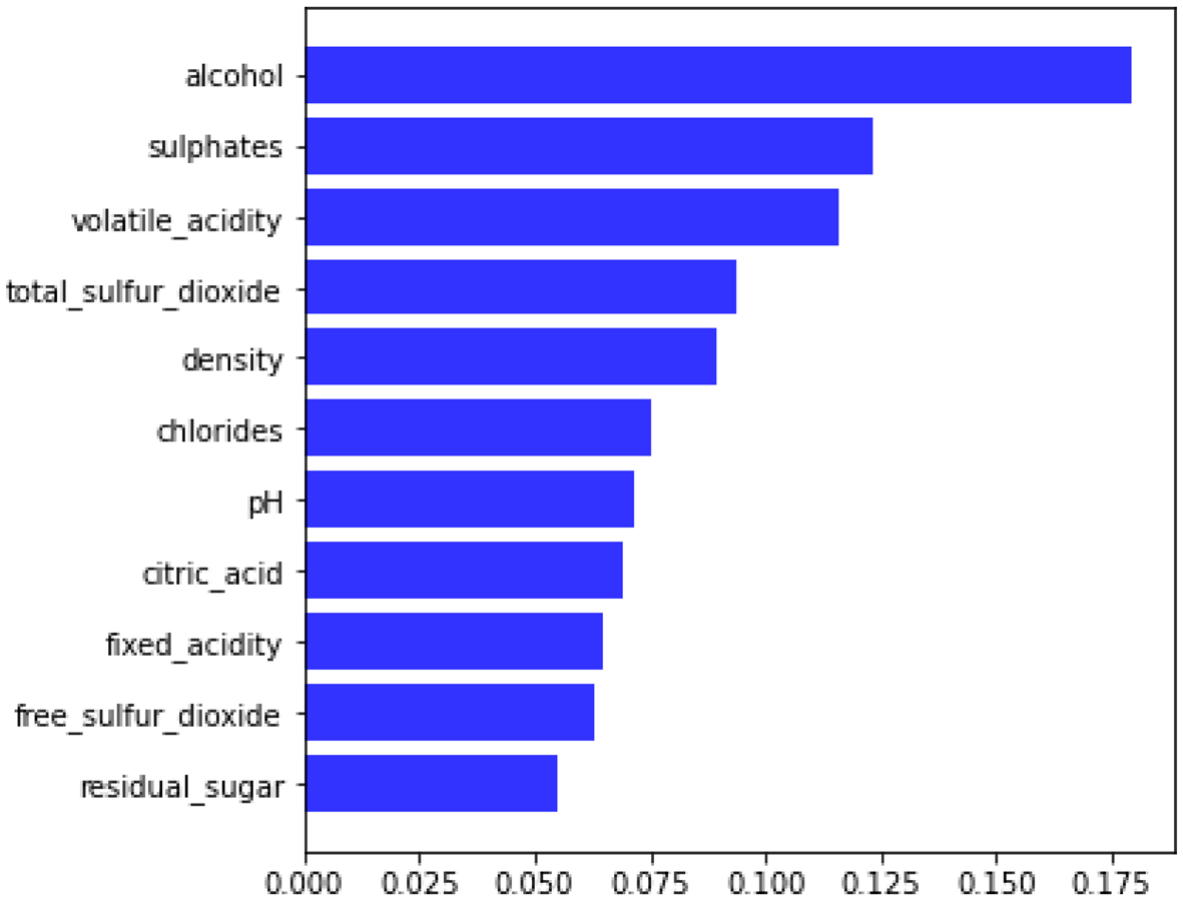
Feature importance of RF on training dataset.

**Figure 16:**
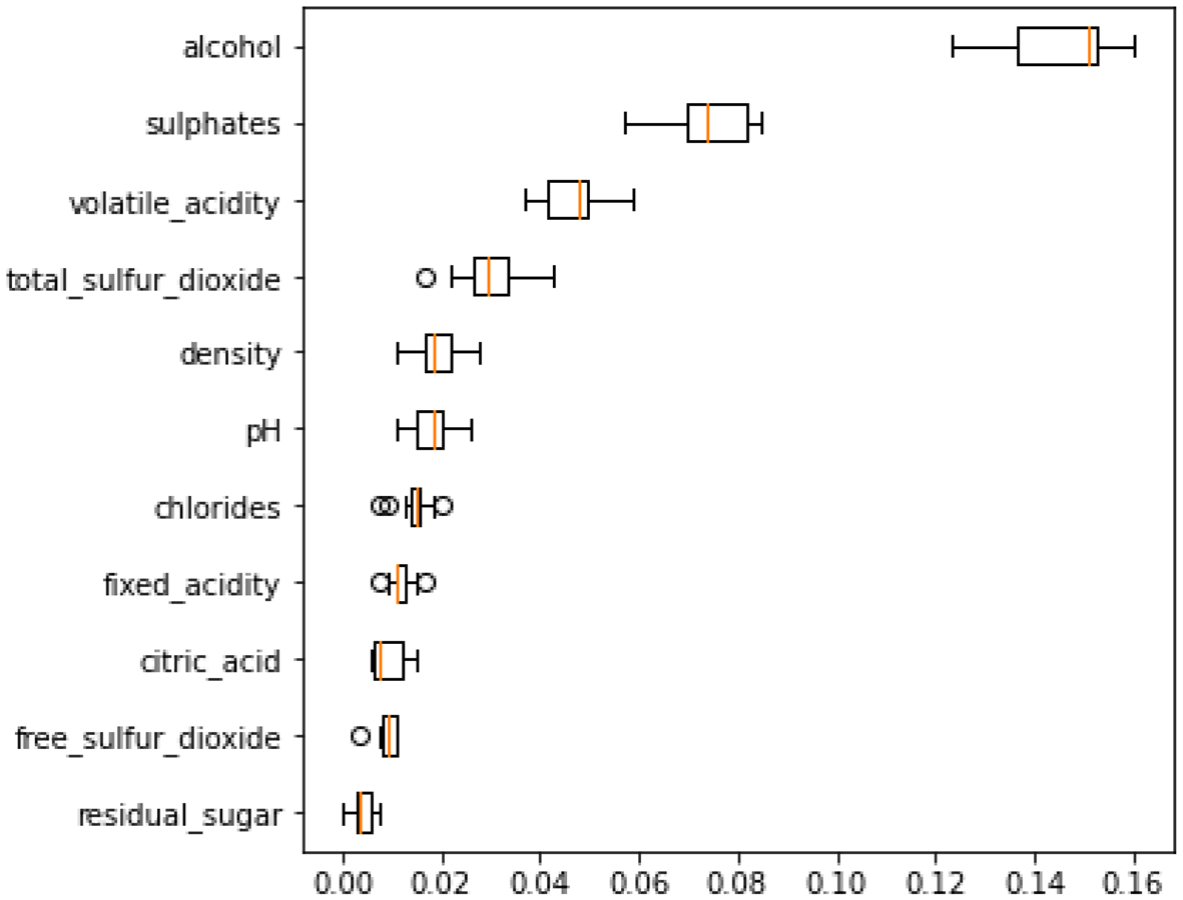
Permutation importance of RF for training dataset.

**Figure 17:**
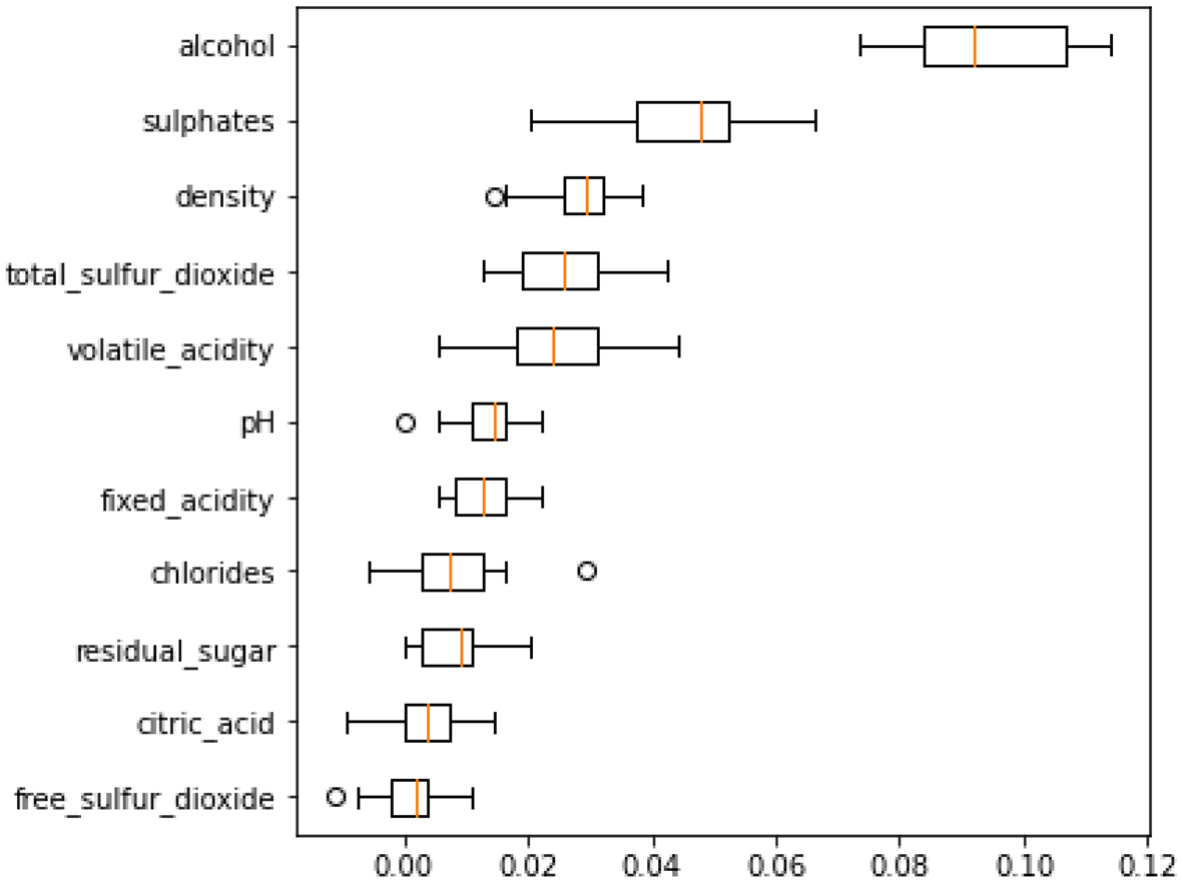
Permutation importance of RF for validation dataset.

Though few ML experts appear to be aware of this, the RF important technique we'll work in this section (permutation importance) applies to any model. Permutation importance is a popular, largely effective, and quite dependable method. Observing the impact of randomly rearranging each predictor variable on the model’s accuracy can determine each variable's relative importance. Because it does not rely on internal model variables like linear regression coefficients, this technique has a wide range of applications. The important feature of RF on the training dataset is illustrated in Fig. 15, which show that the alcohol, sulfates, volatile acidity, and total sulfur dioxide as the top four feature. The permutation importance of RF for the training dataset is represented by boxplots in Fig. 16 and the permutation importance of RF for the validation dataset is represented by boxplots in Fig. 17.

### Clustering analysis

**Figure 18:**
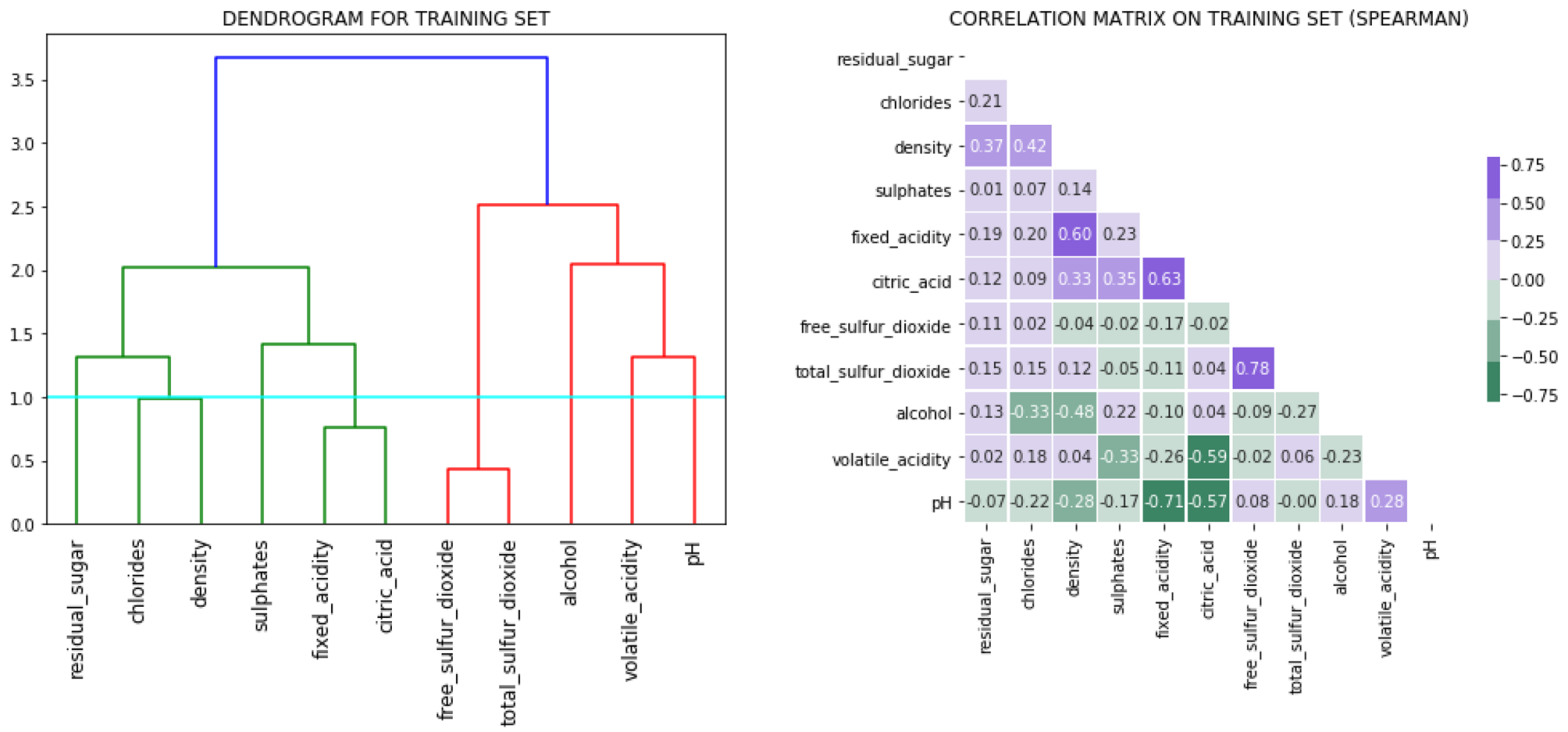
Dendrogram (left) and Spearman’s correlation matrix (right) for the training dataset.

To further investigate the performance of the RF classifier, we computed the wards’ minimum variance criterion on the training dataset. The training dataset was used to create this dendrogram diagram, which shows the hierarchy of relationships between objects. For this work, we used hierarchical clustering. To decide how to allocate items to clusters, a dendrogram is frequently utilised. Figure 18 illustrates the dendrogram for the training dataset (left) and spearman’s correlation matrix on the training dataset (right).

**Figure 19:**
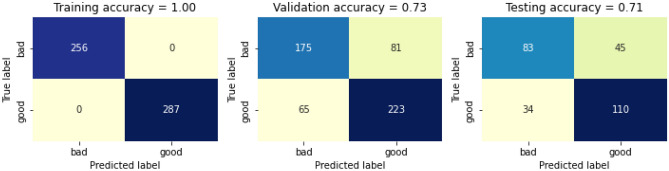
Confusion matric before calibrating the selected eight features.

**Figure 20:**
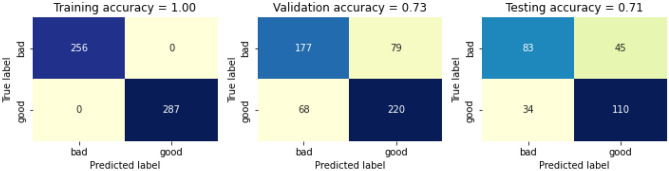
Confusion matrix after calibrating the selected eight features.

Based on the dendrogram and spearman’s correlation matrix, we selected eight features and removed three. The selected eight features are Alcohol, fixed acidity, volatile acidity, residual sugar, chlorides, free sulfur dioxide, pH, and sulfates. The three removed features are citric acid, total sulfur dioxide, and density. The RF classifier is then trained with selected eight features. The confusion matrix before calibrating it with the selected eight features is presented in Fig. 19. The training accuracy before calibrating the swapped dataset is 100%, whereas the validation accuracy and testing accuracy before calibrating are 73% and 73%, respectively, thus giving a total accuracy before calibrating 83%. The confusion matrix after calibrating it with the selected features is presented in Fig. 20. Again, the training accuracy after calibrating the selected feature is 100%, whereas the validation accuracy and testing accuracy before calibrating is 73% and 71%, respectively, thus giving a total accuracy before calibrating is 83%.

**Figure 21:**
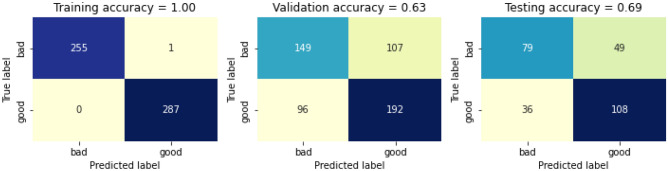
Confusion matric before calibrating the removed three features.

**Figure 22:**
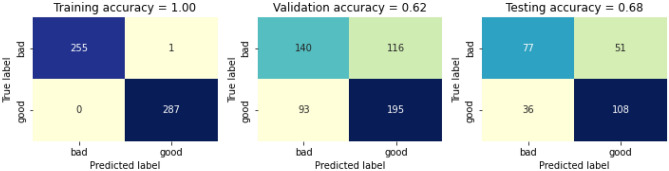
Confusion matrix after calibrating the removed three features.

The RF classifier is then trained with the removed three features. The confusion matrix before calibrating it with the removed features is presented in Fig. 21. The training accuracy before calibrating the swapped dataset is 100%, whereas the validation accuracy and testing accuracy before calibrating is 63% and 69% respectively, thus giving a total accuracy before calibrating is 79%. The confusion matrix after calibrating it with the removed three features is presented in Fig. 22. The training accuracy after calibrating the removed features is 100%, whereas the validation accuracy and testing accuracy before calibrating is 62% and 68% respectively, thus giving a total accuracy after calibrating is 78%.

**Figure 23:**
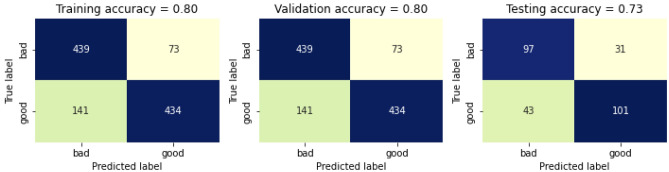
Confusion matrix for selected features with hyperparameter tuning.

The hyperparameter tuning is performed and best parameters found by grid search. The number of estimators is chosen as 50, 75 and 100. The maximum features are selected as 2 and 5 which determines the best split. The maximum depth of the tree id taken as 3, 5 and 7. And the class weight is taken as none, balanced and balanced subsample. The best cross validation score is achieved as 76.45%. The confusion matrix for selected features with hyperparameter tuning is presented in Fig. 23. The training accuracy for selected features with Hyperparameter Tuning is 80%, whereas the validation accuracy and testing accuracy is 80% and 73% respectively, thus giving a total accuracy of 79%.

We determined the RF and XGB classifier to be the best model for predicting wine quality after performing model training and testing under numerous conditions. We also demonstrated the significance of feature selection by enhancing classifier accuracy. We also established how the model's performance was positively impacted by key variables chosen through the feature selection technique. Overall, findings from numerous research relating to wine quality fully concur with those found in this investigation.

## Conclusion

Interest in the wine industry has grown recently, which begs for industrial expansion. To increase wine production and sales, corporations are investing in cutting-edge technologies. For each of these procedures, wine quality certification is essential and necessitates expert human wine testing. We utilised samples from the red wine dataset (RWD) with eleven distinct physiochemical properties. With the initial sample of RWD, five ML models were trained and evaluated. We evaluated the effectiveness of the RF and XGBoost classifiers based on accuracy, recall, F1 scores, and support before introducing them as ML models to predict wine quality. Using these two ML methodologies, the top three features are chosen from a total of eleven features, and ML analysis is performed on the other features. Various plots are used to represent the feature importance based on the XGBoost model and RF. Wine quality was predicted using significant characteristics (also known as essential factors) that were demonstrated to be meaningful in at least three feature selection approaches. When trained and evaluated without feature selection, with feature selection (RF), and with key variables, the XGBoost classifier displayed 100% accuracy (features found important in feature selection). The performance of the RF classifier was improved in the presence of necessary variables. Finally, we have trained an RF classifier, calibrated it, and adjusted its hyperparameters to evaluate the accuracy of its predictions. We also carried out cluster analysis to manage collinearity and limit the number of predictors without compromising model accuracy. Overall, all classifiers performed better when trained and evaluated utilizing key factors. The main aspect of this study is the value of data production techniques and the significance of feature selection. In the future, large datasets can be used for studies, and additional ML and deep learning methods could be investigated for predicting wine quality.

## Data Availability

The datasets generated and/or analysed during the current study are available in the https://archive.ics.uci.edu/ml/datasets/wine+quality repository, https://www.kaggle.com/datasets/uciml/red-wine-quality-cortez-et-al-2009.
